# STK295900, a Dual Inhibitor of Topoisomerase 1 and 2, Induces G_2_ Arrest in the Absence of DNA Damage

**DOI:** 10.1371/journal.pone.0053908

**Published:** 2013-01-22

**Authors:** Sun-Ok Kim, Krisada Sakchaisri, Thimmegowda N. R., Nak Kyun Soung, Jae-Hyuk Jang, Young Sang Kim, Kyung Sang Lee, Yong Tae Kwon, Yukihiro Asami, Jong Seog Ahn, Raymond Leo Erikson, Bo Yeon Kim

**Affiliations:** 1 Korea Research Institute of Bioscience and Biotechnology (KRIBB), Ochang, Cheongwon, Korea; 2 Department of Biochemistry, College of Natural Sciences, ChungNam National University, Yuseonggu, Daejeon, Korea; 3 Laboratory of Metabolism, National Cancer Institute, National Institutes of Health, Bethesda, Maryland, United States of America; 4 World Class University (WCU), Graduate School of Convergence Science and Technology and College of Medicine, Seoul National University, Seoul, Korea; 5 Center for Pharmacogenetics and Department of Pharmaceutical Sciences, School of Pharmacy, Universigy of Pittsburgh, Pittsburgh, Pennsylvania, United States of America; 6 Chemical Biology Department, RIKEN Advanced Science Institute, Wako-shi, Saitama, Japan; 7 Department of Molecular and Cellular Biology, Harvard University, Cambridge, Massachusetts, United States of America; Indiana University School of Medicine, United States of America

## Abstract

STK295900, a small synthetic molecule belonging to a class of symmetric bibenzimidazoles, exhibits antiproliferative activity against various human cancer cell lines from different origins. Examining the effect of STK295900 in HeLa cells indicates that it induces G_2_ phase arrest without invoking DNA damage. Further analysis shows that STK295900 inhibits DNA relaxation that is mediated by topoisomerase 1 (Top 1) and topoisomerase 2 (Top 2) *in vitro*. In addition, STK295900 also exhibits protective effect against DNA damage induced by camptothecin. However, STK295900 does not affect etoposide-induced DNA damage. Moreover, STK295900 preferentially exerts cytotoxic effect on cancer cell lines while camptothecin, etoposide, and Hoechst 33342 affected both cancer and normal cells. Therefore, STK295900 has a potential to be developed as an anticancer chemotherapeutic agent.

## Introduction

Cancer is a multi-step process resulting from acquired genetic and epigenetic alterations that abrogate normal control of cellular functions and eventually lead to uncontrollable cell growth and proliferation [1], [2]. In recent years, the advances in understanding the molecular basis of cancer have led to a significant improvement of diagnostics and therapeutics for a better management of diseases. However, a number of chemotherapeutic agents that exert chemotherapeutic action through their ability to inhibit nuclear DNA topoisomerases (Tops) have been the mainstay of cancer treatment for many decades [3].

Tops are evolutionally conserved nuclear enzymes, which are essential for DNA metabolism where they are involved in generating the necessary topological state of DNA during replication, transcription, recombination, and chromatin remodeling [4], [5]. Tops act by introducing a sequential breakage and rejoining of one DNA strand (Top 1) or both DNA strands (Top 2) allowing DNA to be transformed between topological isoforms. Therefore, these enzymes have been identified as important targets for cytotoxic drugs and their inhibitors are widely used for decades in cancer chemotherapy.

The Top inhibitors can be classified into two classes according to their mechanism of action: Top poisons and catalytic inhibitors [3], [6], [7]. Top poisons, such as camptothecin and etoposide are able to stabilize the covalent complexes between the enzyme and DNA, termed cleavable complex, and prevent the rejoining step of the reaction thereby resulting in accumulation of DNA strand break. Consequently, tumor cell death is triggered by the substantial DNA damage evoked by Top poisons [8], [9]. On the other hand, the catalytic inhibitors act on any of the other steps in the catalytic cycle by preventing the binding between Top and DNA (aclarubicin) or interfering with the binding or release of ATP (novobiocin, ICRF-193), resulting in activating the decatenation checkpoint [7], [10], [11].

We report here a symmetric bibenzimidazole derivative, STK295900, as a Top catalytic inhibitor. STK295900 efficiently inhibited the growth of various cancer cell lines such as HeLa, MCF7, HepG2, and HL-60. In addition, cells treated with STK295900 were arrested in G_2_ phase without activation of DNA damage checkpoint. These findings may therefore suggest a potential development of symmetric bibenzimidazole as a chemotherapeutic agent.

## Materials and Methods

### Materials

Dulbecco’s modified Eagle’s medium (DMEM), RPMI 1640, and DMEM/F12 were purchased from HyClone (Logan, UT). Fetal bovine serum (FBS) was purchased from Invitrogen (San Diego, CA). ICRF-193 was obtained from Enzo Life science (Farmingdale, NY). Camptothecin, etoposide, nocodazole, and β-actin antibody were purchased from Sigma-Aldrich (St. Louis, MO). Phospho-Cdk1 (T14), phospho-Cdk1 (Y15), phospho-Cdk1 (T161), Cdk1, cyclin B1, phospho-ATM (S1981), ATM, phospho-ATR (S428), ATR, phospho-Chk1 (S345), Chk1, phospho-Chk2 (T68), Chk2, phospho-Histone H3 (S10), Histone H3, and γ-H2A.X (S139) antibodies were purchased from Cell Signaling Technology (Denvers, MA). Cyclin A, Wee1, Cdc25C, p53, p21, and GAPDH antibodies were purchased from Santa Cruz Biotechnology (Santa Cruz, CA).

### Cell Culture and Cell Growth Inhibition Assay

Human epitheloid cervical carcinoma HeLa cells, human breast adenocarcinoma MCF7 and MDA-MB-231cells, human hepatocellular carcinoma HepG2 cells, human colon adenocarcinoma HT-29 cells, and human colorectal carcinoma HCT-116 cells were purchased from ATCC® and maintained in DMEM medium. Human prostate carcinoma PC-3 cells (purchased from ATCC®), human gastric carcinoma SNU-484 and SNU-601(obtained from Korean Cell Line Bank (KCLB)), human leukemia K-562 and HL-60 cells (purchased from ATCC®), immortalized human prostate epithelial 267B1cells [12], and immortalized human embryonic lung fibroblast MRC5CV1 cells [13] were maintained in RPMI 1640 medium whereas immortalized retinal pigment epithelial hTERT RPE-1 cells (purchased from ATCC®) were maintained in DMEM/F12 medium. All cell lines were cultured in media supplemented with 10% fetal bovine serum, 100 U/ml penicillin, and 100 mg/ml streptomycin at 37°C in a 5% CO_2_ incubator.

Cells were seeded at the appropriate amount (1–2×10^3^ cells) in triplicate in 96-well plates for 24 h and then treated with various concentrations of STK295900. Cell growth was determined by using EZ-CyTox cell viability assay kit (Daeil Lab. Service, Seoul, Korea). Ten microliters of WST solution were added to the treated cells at 24, 48, 72, and 96 h. Cells were further incubated at 37°C for 2 h and the absorbance was measured at 450 nm.

### Flow Cytometric Analysis

For cell cycle analysis, HeLa cells were harvested following compound treatment for 24 h and stained with propidium iodide (PI) according to the instruction of Cycle Test Plus DNA Reagent kit from BD Biosciences (San Jose, CA). Flow cytometric analysis was performed using a FACSCalibur instrument (Becton Dickinson, San Jose, CA).

### Supercoiled DNA Relaxation Assay for Topoisomerase 1

The relaxation assay for topoisomerase 1 was performed using purified calf thymus topoisomerase 1 and pBR322 plasmid DNA (Takara Bio, Shiga, Japan). In brief, 0.25 µg of pBR322 DNA was incubated with 1 unit of topoisomerase 1 in 20 µl reaction containing 35 mM Tris-HCl pH 8.0, 72 mM KCl, 5 mM MgCl_2_, 5 mM DTT, 5 mM spermidine, and 0.1% BSA. The mixture was incubated for 30 min at 37°C in the presence or absence of STK295900 and camptothecin. The reaction was terminated by addition of 2 µl 10% SDS and then treated with 50 µg/ml proteinase K for 30 min at 37°C to digest the protein. Samples were resolved by electrophoresis on 1% agarose gel. After gel electrophoresis, the gel was stained with ethidium bromide and DNA bands were visualized by UV light and photographed using Gel Doc XR (Bio-Rad, Hercules, CA).

### Supercoiled DNA Relaxation Assay for Topoisomerase 2α

The relaxation assay for topoisomerase 2α was performed in 20 µl reaction mixture containing 0.25 µg of plasmid pBR322 DNA in DNA topoisomerase 2 buffer (50 mM Tris-HCl pH 8.0, 150 mM NaCl, 10 mM MgCl_2_, 2 mM ATP, and 0.5 mM DTT) and 1 unit of human topoisomerase 2α in the absence or presence of STK295900, etoposide, or ICRF-193 for 30 min at 37°C. After incubation, the reaction was terminated by addition of 2 µl of 10% SDS. The reaction mixtures were treated with 50 µg/ml proteinase K for 30 min at 37°C and then DNA was extracted with CIA (chloroform:isoamyl alcohol, 24∶1). Samples were resolved by electrophoresis on a 1% agarose gel. After staining the electrophoresed gel with ethidium bromide, DNA bands were visualized by UV light and photographed on Gel Doc XR.

### Immunofluorescence Cell Staining

For phospho-Histone H2A.X (S139) staining, HeLa cells were cultured in black 96-well plate for 24 h. Cells were then treated with STK295900, ICRF-193, etoposide, and camptothecin for another 24 h. The treated cells were fixed with 3.7% formaldehyde for 20 min, washed once with PBS, and permeabilized by 0.2% Triton X-100 in PBS for 5 min at room temperature. After blocking the samples with 5% BSA in PBS for 30 min at room temperature, the cells were incubated sequentially with the phospho-Histone H2A.X (S139) antibody (1∶50 dilution in 0.02% Triton X-100 and 0.1% BSA in PBS) for 90 min at 37°C and then with Alexa-conjugated secondary antibody (1∶200 dilution in PBS containing 0.1% BSA) for 60 min at 37°C. DNA of ICRF-193-, etoposide-, and camptothecin-treated cells was stained with 0.5 µg/ml Hoechst 33342 in PBS. Images were analyzed on a fluorescence microscope (Nikon Instruments Inc., Melville, NY).

### Immunoblot

Cells were lysed with cold RIPA buffer (50 mM Tris-HCl pH 8.0, 150 mM NaCl, 1% NP-40, 0.5% sodium deoxycholate, 0.1% SDS) containing freshly added protease inhibitor, phosphatase inhibitor (SIGMA), 1 mM DTT, and 1 mM Na_3_VO_4_ on ice for 1 h. Whole cell lysates were centrifuged at 15,000 rpm for 20 min and then the supernatant was transferred into a new tube. An appropriate amount of cell lysate was subjected to SDS-PAGE. Then the resolved proteins were transferred onto a PVDF membrane (Bio-Rad) for immunoblot analyses with specific antibodies. The proteins were detected using chemiluminescence solution (Thermo Scientific, Rockford, IL).

## Results

### Effect of STK295900 on Various Human Cancer Cells

**Figure 1:**
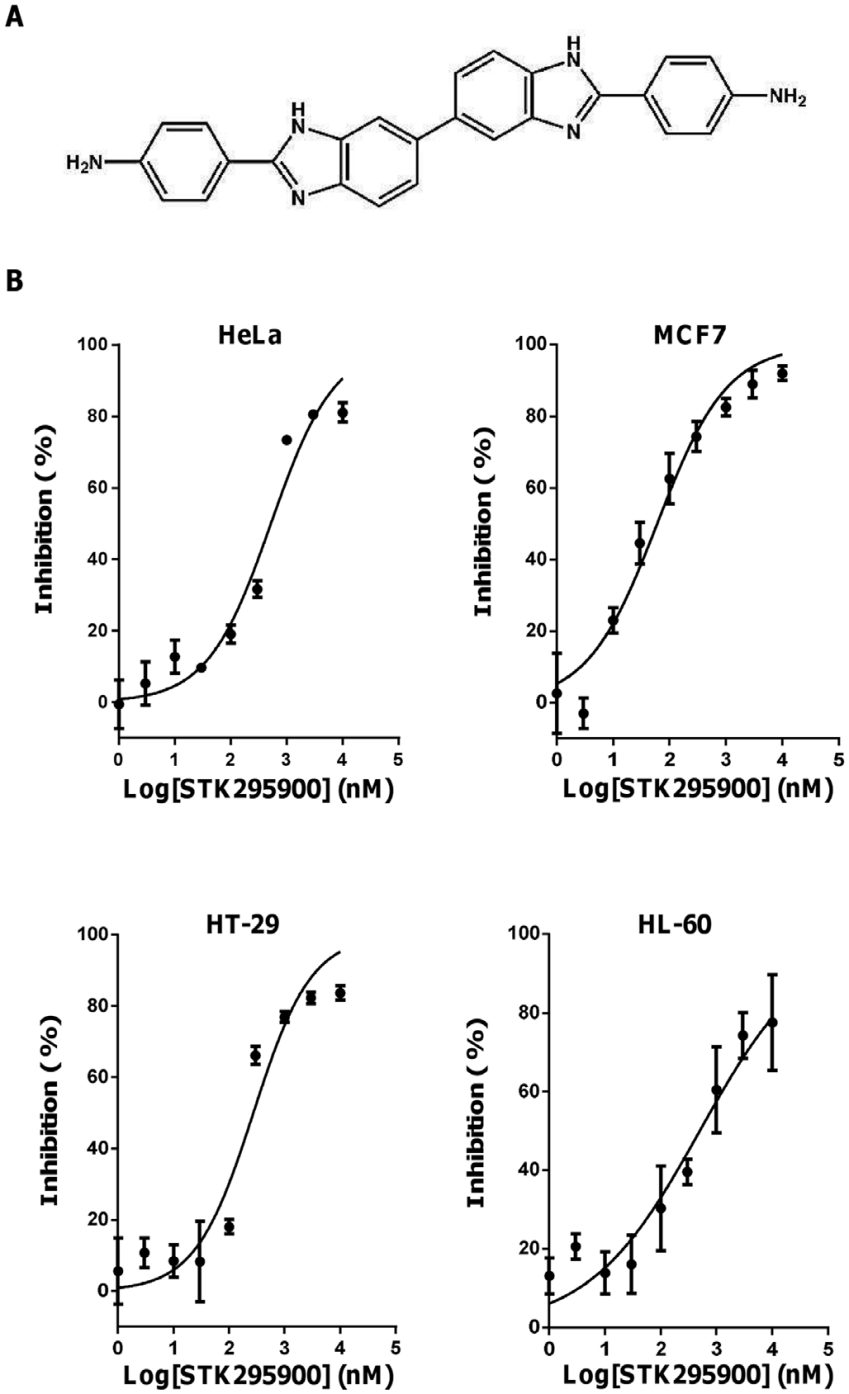
Effects of STK295900 on proliferation of various human cancer cell lines. (A) Chemical structure of STK295900. (B) Inhibitory effects of STK295900 on the proliferation of variety of cancer cell lines. Cells were seeded at 1−2×10^3^ cells in 96 well plates and treated with various concentrations of STK295900 for 4 days. Cell growth was determined by MTT assay. Data were fitted with dose-response curve using Graphpad Prism software. Values represent the mean ± SD from a representative triplicate experiment.

In our effort to identify new antiproliferative compounds from small molecule library, we found a symmetric bibenzimidazole derivative STK295900 (4,4′-(3H,3′H-5,5′-bibenzimidazole-2,2′-diyl)dianiline) (Fig. 1A) that strongly inhibited the growth of various cancer cell lines. As shown in Fig. 1B, STK295900 efficiently suppressed the growth of HeLa, MCF7, HT-29, and HL-60 cells in a dose-dependent manner with IC_50_ values 0.52, 0.06, 0.27, and 0.50 µM, respectively (Table 1). Furthermore, STK295900 also inhibited the growth of the other cancer cell lines including MDA-MB-231, HepG2, HCT-116, K-562, PC-3, SNU-484, and SNU-601 cells, with IC_50_ ranging from 0.51–2.83 µM (Table 1). These results indicate that STK295900 exhibits antiproliferative effect against a variety of cancer cell types.

**Table 1:** Inhibitory effect of STK295900 on proliferation of various cancer cell lines.

Tumor	Cell Line	IC_50_ (µM)
Cervical carcinoma	HeLa	0.52
Breast adenocarcinoma	MCF7	0.06
	MDA-MB-231	0.51
Hepatocellular carcinomar	HepG2	0.17
Colon adenocarcinoma	HT-29	0.27
	HCT-116	2.83
Leukemia	HL-60	0.50
	K-562	1.38
Prostate carcinoma	PC-3	2.72
Gastric carcinoma	SNU-484	1.21
	SNU-601	2.21

Cells were seeded at 1−2×10^3^ cells in 96 well plates and treated with various concentrations of STK295900. Cell growth was determined by MTT assay for up to 4 days. All experiments were done in triplicates and IC_50_ was calculated from log-dose-response curves.

Moreover, we also compared the cytotoxic effects of STK295900, Top 1 inhibitors (camptothecin and Hoechst 33342), and Top 2 inhibitor (Etoposide) in various cell lines including cancer (HeLa, MCF7, HepG2, and HT-29) and non-cancerous cell lines (hTERT RPE-1, 267B1, and MRC5CV1). Camptothecin exhibited strong cytotoxicity towards all cell lines tested with IC_50_ less than 30 nM while etoposide also showed high cytotoxicity against those cells except MCF7 and HT-29 (IC_50_ 13.21 and 10.26 µM, respectively) (Table 2). The effect of Hoechst 33342 was diverse and non-selective against cancer cells (Table 2). In contrast, STK295900 exhibited selective toxicity against cancer cells (IC_50_s 0.64, 0.04, 0.14, and 0.21 µM in HeLa, MCF7, HepG2, and HT-29, respectively) when compared to non-cancer cells (IC_50_s 3.43, 1.61, and 0.95 µM in hTERT RPE-1, 267B1, and MCR5CV1, respectively) (Table 2).

**Table 2:** Comparison of STK295900 with camptothecin, etoposide, and Hoechst 33342 for its effect on the proliferation of various cancer and non-cancer cell lines.

		IC_50_ (µM)		
Cell Line	STK295900	Camptothecin	Etoposide	Hoechst 33342
HeLa	0.64	0.02	0.30	4.56
MCF7	0.04	0.03	13.21	0.20
HepG2	0.14	0.02	0.84	0.22
HT-29	0.21	0.03	10.26	0.65
hTERT RPE-1	3.43	<0.01	0.45	1.01
267B1	1.61	<0.01	0.39	0.84
MRC5CV1	0.95	0.02	1.29	0.35

Cells were seeded at 1−2×10^3^ cells in 96 well plates and treated with various concentrations of STK295900, camptothecin, etoposide, or Hoechst 33342. Cell growth was determined by MTT assay for up to 4 days. All experiments were done at least in triplicates, and IC_50_ was calculated from dose-response curves.

### STK295900 Induces G_2_ Phase Arrest

**Figure 2:**
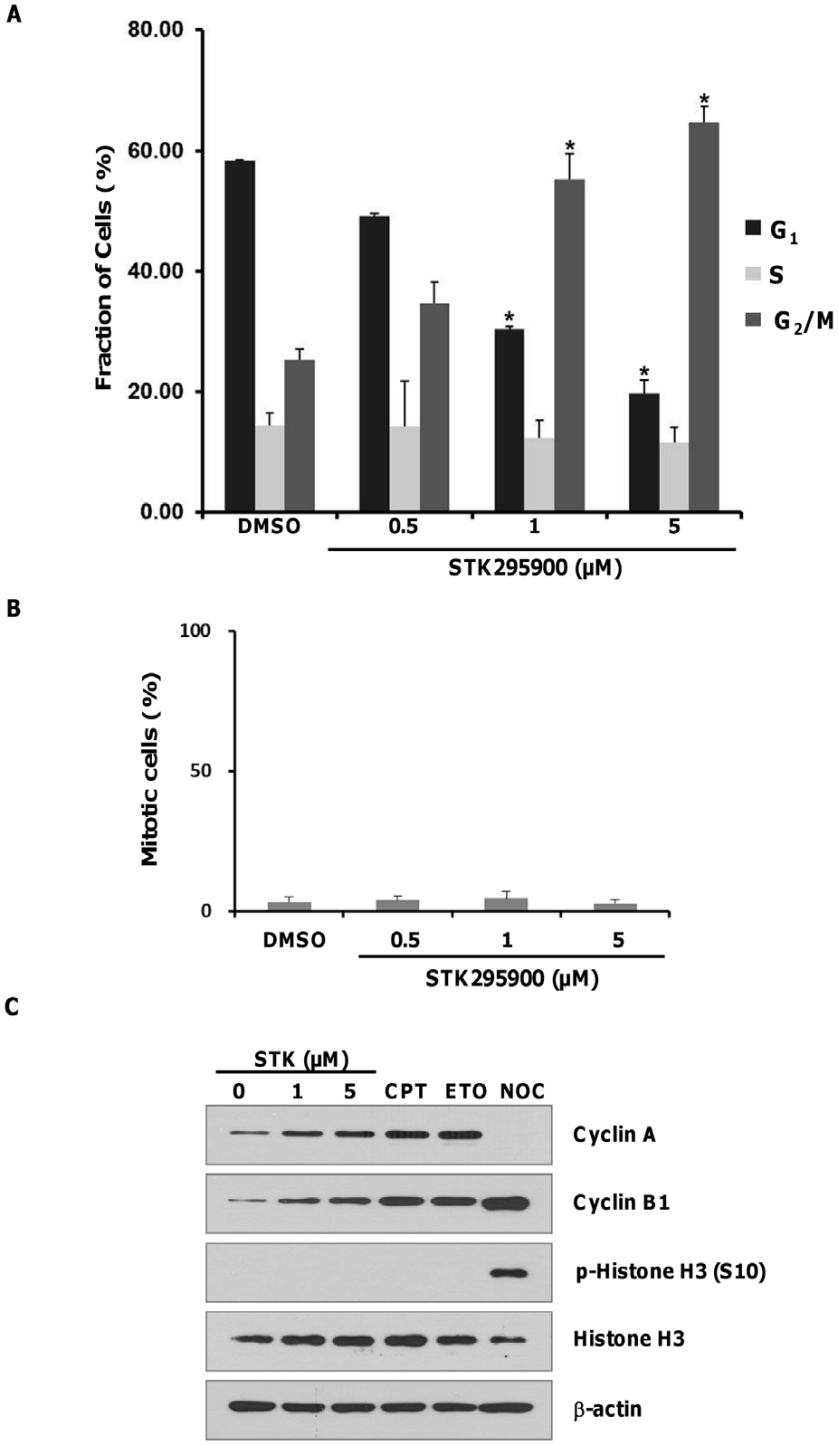
STK295900 induces G_2_ phase arrest in HeLa cells. (A) Flow cytometric analysis for cell cycle distribution. HeLa cells were treated with the indicated concentrations of STK295900 for 24 h. Treated cells were then stained with propidium iodide (PI) and processed for cell cycle analysis. The bar graph represents the mean percentage of each cell cycle phase ± SD from three independent experiments. * = p<0.05 versus the respective G_1_, S, or G_2_/M phase of DMSO-treated cells. (B) Mitotic index of STK295900. HeLa cells were treated with the indicated concentrations of STK295900 for 24 h. Cells were then stained with Hoechst 33342 and mitotic cells were counted. The bar graph shows mean ± SD from the representative of triplicate experiments. (C) Cell cycle related proteins expression. HeLa cells were treated with DMSO control, STK295900 (STK) 1 or 5 µM, camptothecin (CPT) 10 µM, etoposide (ETO) 10 µM, or nocodazole (NOC) 200 ng/ml for 24 h. Treated cells were lysed and subjected to immunoblot analyses with antibodies against cyclin A, cyclin B1, phospho-Histone H3 (S10), and Histone H3. β-actin was used as a loading control.

Given its strong growth inhibitory effect on various cancer cell types, STK295900 was examined to determine its effect on cell cycle distribution using flow cytometry analysis. As shown in Fig. 2A, around 25% of HeLa control cells were in G_2_/M phase with 4N DNA content. Treatment with STK295900 at 0.5, 1, and 5 µM for 24 h resulted in increased G_2_/M population to about 35%, 55%, and 65%, respectively. This result suggested that STK295900 could induce G_2_/M phase arrest. We then analyzed whether the increasing G_2_/M population in Fig. 2A is indeed G_2_ or M phase by determining the mitotic index and investigating the cell cycle regulatory proteins. To determine mitotic index, the treated cells were stained with Hoechst 33342 and then mitotic cells were counted. However, we observed no significant change in mitotic index after treatment with various concentrations of STK295900 (Fig. 2B) suggesting that STK295900 might cause cell cycle arrest at G_2_ phase.

To confirm the G_2_ arrest effect of STK295900, we then investigated cell cycle related proteins including cyclin A, cyclin B1, and Histone H3 phosphorylation. Camptothecin, etoposide, and nocodazole were used as controls for G_2_ and M phases. Camptothecin and etoposide inhibit Top 1 and Top 2 activities, respectively, thereby inducing G_2_ arrest whereas nocodazole causes microtubule depolymerization resulting in mitotic arrest [14]–[16]. It has been well established that cyclin A and cyclin B1 levels are altered through the cell cycle [17]. The level of cyclin A was increased during S and G_2_ phases but declined in mitosis while cyclin B1 was made at S phase and reached the maximum level at M phase. Treatment of HeLa cells with STK295900, camptothecin, and etoposide for 24 h led to accumulations of cyclin A and cyclin B1 (Fig. 2C). In contrast, nocodazole treatment resulted in mitotic arrest with high level of cyclin B1 and undetectable level of cyclin A (Fig. 2C). Furthermore, Histone H3 phosphorylation at S10, a well-known mitotic marker [18], was detected only in cells treated with nocodazole but not with STK295900, camptothecin, or etoposide. Taken together, these data indicated that STK295900 induced G_2_ arrest.

### Effect of STK295900 on Cdk1 Phosphorylation

**Figure 3:**
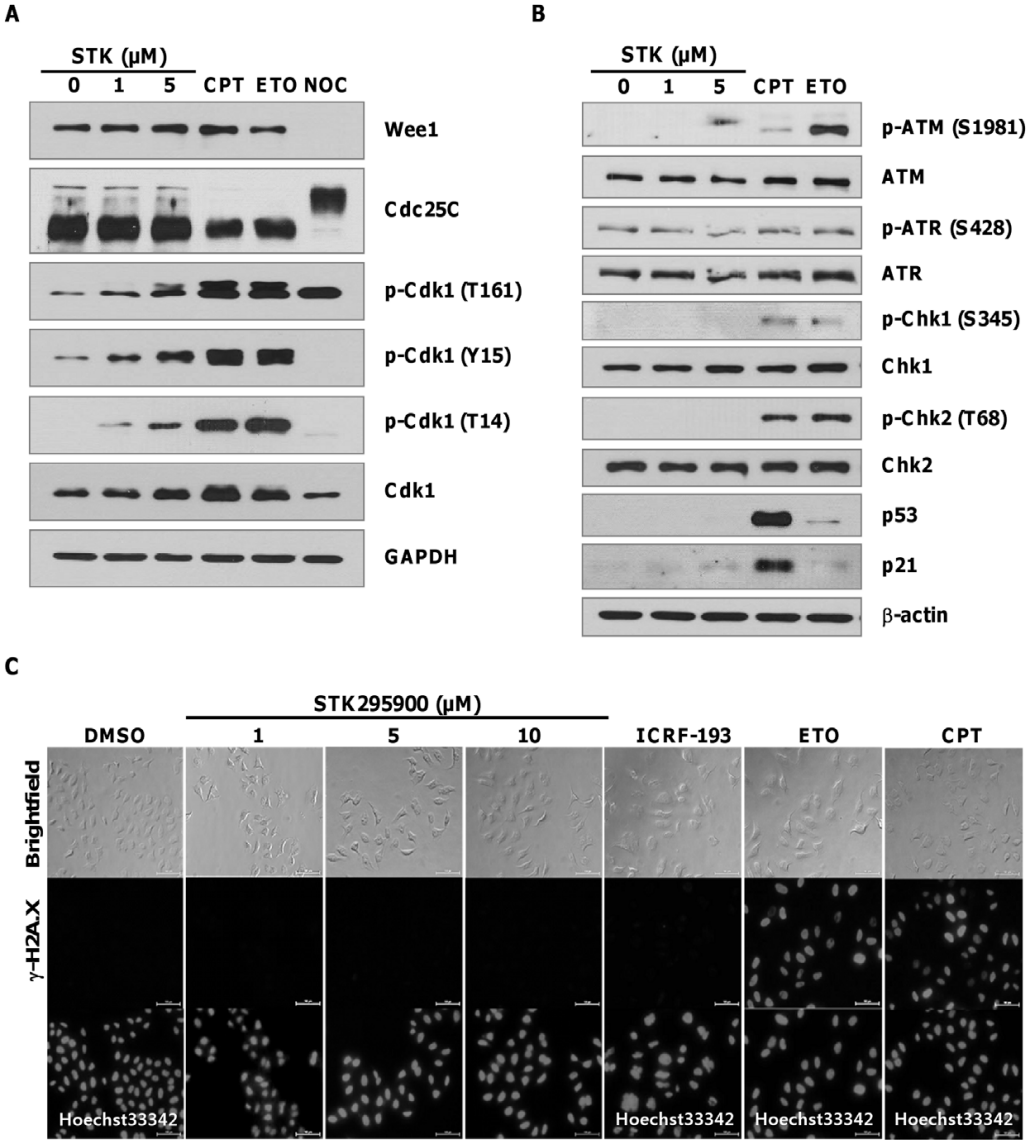
STK295900 does not activate DNA damage checkpoint. (A) G_2_/M transition regulated proteins expression. HeLa cells were treated with STK295900 1 or 5 µM, Camptothecin 10 µM, etoposide 10 µM, or nocodazole 200 ng/ml. After 24 h incubation, cell lysates were prepared for immunoblot analyses with antibodies against phospho-Cdk1 (T161), phospho-Cdk1 (T14), phospho-Cdk1 (Y15), Cdk1, Wee1, and Cdc25C. GAPDH was used as a loading control. (B) DNA damage-checkpoint related proteins. The same lysates used in (A) were subjected to immunoblot analyses with antibodies against phospho-ATM (S1981), ATM, phospho-ATR (S428), ATR, phospho-Chk1 (S345), Chk1, phospho-Chk2 (T68), Chk2, p53, and p21. β-actin was used as a loading control. (C) Immunofluorescence staining for γ-H2A.X. HeLa cells were treated with 1, 5, or 10 µM of STK295900 or 10 µM of ICRF-193, etoposide, and camptothecin for 24 h. Treated cells were then fixed and stained with anti-γ-H2A.X (middle panel). DNA from ICRF-193-, etoposide-, and camptothecin-treated cells was stained with Hoechst 33342 (bottom panel). Images were analyzed on a fluorescence microscope.

In addition to cyclin B1 binding, Cdk1 activity also requires phosphorylation at T161 in its activation loop. Nonetheless, the activity of Cdk1 is kept in check by inhibitory phosphorylations at Y15 and T14 by Wee1 and Myt1, respectively [19], [20]. We then investigated the phosphorylation state of Cdk1 at 24 h after treatment with STK295900, camptothecin, etoposide, and nocodazole. Phosphorylation of Cdk1 at T161 was strongly enhanced in cells treated with camptothecin, etoposide, and nocodazole (Fig. 3A). In contrast, the inhibitory phosphorylation (T14 and Y15) could not be detected in nocodazole-treated cells but was abundance in camptothecin- and etoposide-treated cells (Fig. 3A). STK295900 treatment displayed dose-dependent increase in Cdk1 phosphorylations at T14, Y15, and T161, but the signal intensities were somewhat weaker than in cells treated with camptothecin and etoposide (Fig. 3A). However, treatment of STK295900 in HeLa cells also caused accumulation of Cdk1 whose level was comparable to the increased in its phosphorylation level.

Moreover, we also observed no change in Wee1 and Cdc25C levels in STK295900-treated cells while camptothecin and etoposide treatments caused reduction of Cdc25C (Fig. 3A). Unlike STK295900, nocodazole treatment resulted in undetectable levels of Wee1 but activation of Cdc25C as indicated by retarded migration on SDS-PAGE (Fig. 2C), reflecting its hyperphosphorylation at G_2_/M transition [21]. Taken together, these results suggested that STK295900-induced G_2_ arrest is unlikely due to suppression of Cdk1 activity by inhibitory phosphorylations at T14 and Y15.

### STK295900 does not Activate DNA Damage Checkpoint

Many widely used chemotherapeutic agents cause DNA damage by targeting DNA or enzymes that regulate DNA topology resulting in DNA damage induced G_2_ arrest [14], [22]. DNA damage leads to activation of ATM/ATR signaling pathway [23]. Therefore we investigated whether the G_2_ arrest induced by STK295900 is due to DNA damage checkpoint activation by analyzing the phosphorylation-dependent activation of ATM (S1981), ATR (S428), Chk1 (S345), and Chk2 (T68). As shown in Fig. 3B, treatment with camptothecin and etoposide resulted in activation of ATM, Chk1, and Chk2 as judged by the increased phosphorylation. However, no increase in phosphorylations of ATM, ATR, Chk1, and Chk1 were observed in STK295900-treated cells (Fig. 3B). In addition, while p53 and p21 levels were only weakly upregulated in etoposide-treated cells, they were significantly increased in camptothecin-treated sample (Fig. 3B). Interestingly, however, STK295900 did not induce upregulation of p53 but marginally affected p21 level (Fig. 3B).

To confirm that STK295900 did not induce DNA strand break, we then measured Histone H2A.X phosphorylation at S139 (γ-H2A.X), a hallmark of DNA strand break in cells [24]. HeLa cells were treated with 1, 5, or 10 µM of STK295900. Top poisons (etoposide and camptothecin) and Top catalytic inhibitor (ICRF-193) were used as controls. After treatment for 24 h, cells were subjected to immunostaining with anti-γ-H2A.X. As shown in Fig. 3C, STK295900, like ICRF-193, did not induce γ-H2A.X signal while Top poisons etoposide and camptothecin strongly induced it (Fig. 3C). Taken together, these data indicated that G_2_ arrest induced by STK295900 was irrelevant to DNA damage. Furthermore, we also observed that STK295900 could stain DNA and be also excited by ultraviolet light to emit blue fluorescence similar to Hoechst 33342 (Fig. 3C) suggesting that STK295900 bind to DNA and therefore might exert its effect through this mechanism.

### STK295900 Inhibits Tops Activities

**Figure 4:**
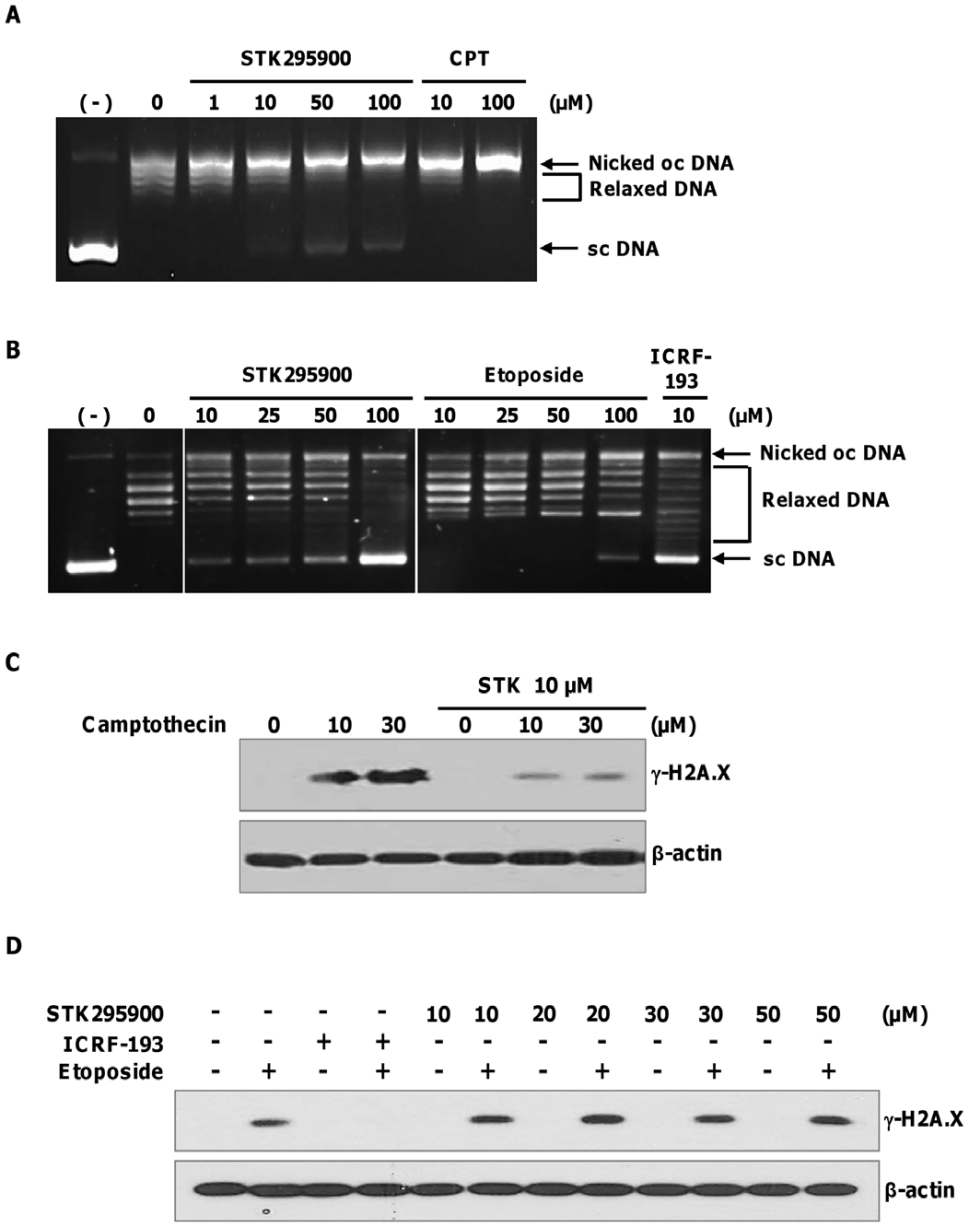
STK295900 inhibits topoisomerases activities. Supercoiled DNA relaxation assay for (A) topoisomerase 1 (Top 1) and (B) topoisomerase 2 (Top 2). Supercoiled pBR322 plasmid DNA was incubated at 37°C for 30 min with Top 1 enzyme (A) or Top 2α (B) in the presence of various concentrations of indicated compounds. DNA samples were separated by electrophoresis on a 1% agarose gel, stained with ethidium bromide, and visualized by UV light. (−), supercoiled DNA alone; oc, open circular; sc, supercoiled. (C) Antagonistic effect of STK295900 in camptothecin-induced DNA damage. HeLa cells were pretreated with 10 µM of STK295900 for 30 min and then incubated with the indicated concentrations of camptothecin for 1 h. Treated cells were lysed and subjected to immunoblot analyses with antibody against γ-H2A.X. β-actin was used as a loading control. (D) Antagonistic effect of STK295900 on etoposide-induced DNA damage. HeLa cells were pretreated with STK295900 at 10, 20, 30, or 50 µM or ICRF-193 at 10 µM for 30 min. Cells were incubated with 10 µM of etoposide for another 1 h. Treated cells were then lysed and subjected to immunoblot analyses with antibody against γ-H2A.X. β-actin was used as a loading control.

Many DNA-binding compounds exhibit their major pharmacological effect through interference with the activity of Tops [25]. Therefore, we firstly investigated the effect of STK295900 on Top 1-mediated DNA relaxation. Top cleaves supercoiled DNA and thereby converts it to less-supercoiled form [4]. DNA relaxation assay was performed using purified Top 1 in the presence of various concentrations of STK295900. As shown in Fig. 4A, STK295900 as well as camptothecin (Top 1 poison) inhibited DNA relaxation activity of Top 1 in a dose-dependent manner as judged by a decrease in relaxed DNA and an increase in nicked-open-circular DNA due to stabilization of the cleavage complex. However, supercoiled DNA could be observed in samples treated with high concentrations of STK295900 (50 and 100 µM), but not with camptothecin, indicating that STK295900 at high concentration may also inhibit Top 1 catalytic activity *in vitro*.

In addition, we also analyzed the effect of STK295900 on Top 2α-mediated DNA relaxation. Etoposide (Top 2 poison) and ICRF-193 (Top 2 catalytic inhibitor) were used as controls according to their activities. Fig. 4B demonstrated that etoposide inhibited Top 2α activity by reducing the relaxed DNA and increasing nicked-open-circular DNA. In contrast, STK295900 also inhibited Top 2α-mediated DNA relaxation in a manner similar to ICRF-193 resulting in accumulation of supercoiled DNA (Fig. 4B). Taken together, these data indicated that STK295900 inhibits both Top 1 and Top 2 activities *in vitro*.

However, as shown in Fig. 3C, STK295900 did not induce DNA strand break associated γ-H2A.X signal, suggesting that it functions as Top catalytic inhibitor. To determine antagonistic effect of STK295900 on Top poison-mediated DNA damage, HeLa cells were pretreated with DMSO or STK295900 for 30 min and then incubated with 10 µM or 30 µM of camptothecin or etoposide for 1 h. The lysates were subjected to immunoblot analyses with γ-H2A.X antibody. In control cells, camptothecin and etoposide treatment strongly induced γ-H2A.X (Figs. 4C & 4D). STK295900 pretreatment dramatically reduced camptothecin-induced γ-H2A.X (Fig. 4C). Interestingly, however, STK295900 up to 50 µM, could not prevent etoposide-induced γ-H2A.X (Fig. 4D). These results indicated that STK295900 antagonizes Top 1 poison-mediated DNA damage.

## Discussion

In the search for new chemotherapeutic agents from the small molecule library, we identified STK295900 (Fig. 1A) that exhibited efficient antiproliferative activity against various cancer cell lines of different origin, especially MCF7 and HepG2 (Fig. 1B and Table 1). Furthermore, analyzing the effect of STK295900 on HeLa cells demonstrated that it induced G_2_ cell cycle arrest (Fig. 2). This is due to STK295900-induced accumulation of 4N DNA content with no significant change in mitotic index (Figs. 2A and 2B). In addition, STK295900-induced G_2_ arrest was confirmed by investigating the cell cycle regulatory proteins. Progression through the eukaryotic cell cycle is driven in part by a subfamily of Cdks whose activities are modulated by forming bipartite complexes with different cyclins [26]. Levels of cyclins oscillate throughout the cell cycle whereas Cdk protein levels remain stable [27], [28]. Therefore, the activity of Cdks is regulated by the presence of different cyclins. In mitotic cells, cyclin B1 level was relatively high whereas cyclin A was undetectable (Fig. 2C). In contrast, STK295900 showed similar effect as camptothecin and etoposide did on cyclin A and cyclin B1 accumulation without induction of Histone H3 phosphorylation at S10 (Fig. 2C), which is crucial for chromosome condensation and cell-cycle progression during mitosis [18], [29].

STK295900 belongs to a class of symmetric bibenzimidazole group. Compounds containing benzimidazole ring have been used extensively for pharmacological purposes such as antimicrobial and anticancer agents [30]. Several asymmetric, head-to-tail bibenzimidazole derivatives, such as Hoechst 33258 and Hoechst 33342, exhibited antitumor activity by binding to minor groove of DNA at three consecutive A:T base pairs, leading to the inhibition of Top 1 activity [31], [32]. In addition, the symmetric bibenzimidazole derivatives, containing two groups of benzimidazole linked in head-to-head fashion, have been reported that they bind DNA minor groove with extending the binding site to four A:T base pairs and exhibit antitumor activity [33]. However, there is no report on the mechanism of action for their antitumor activity. Here, we showed that STK295900 exerted its activity by interfering with Top 1 and Top 2 activities (Fig. 4). In support of this notion, STK295900 was recently reported as a potent anti-staphylococcal agent by targeting DNA gyrase [34]. The results from DNA relaxation assay suggested that STK295900 stabilizes the DNA-Top 1 cleavable complex, a characteristic of Top poisons (Fig. 4A), but it also inhibited Top 2 catalytic activity (Fig. 4B).

Generally, Top poisons causes DNA strand break and consequently triggers G_2_ arrest via activation of ATM/ATR signaling pathway [3], [6], [23], [35]. These kinases phosphorylate and activate Chk1 and Chk2, which in turn phosphorylate and inactivate Cdc25C phosphatase resulting in blocking the activation of Cdk1 and transition into mitosis [36]–[39]. They also phosphorylate p53 leading to its accumulation and activation resulting in increased transcription of cell cycle arrest-related genes such as p21^CIP^, GADD45, and 14-3-3δ [40], [41]. Moreover, Histone H2A.X becomes locally phosphorylated by ATM/ATR at the vicinity of DNA strand break to generate γ-H2A.X, a well-known marker for DNA strand break [42], [43]. In agreement with γ-H2A.X signal (Fig. 3C), STK295900 also did not trigger DNA damage checkpoint pathway (Fig. 3B). Furthermore, STK295900 showed protective effect against DNA damage induced by camptothecin but not by etoposide (Fig. 4C). Thus, STK295900 at physiological concentration may prevent the binding of Top 1 to DNA and, as a consequence, prevent Top 1 poison-induced DNA damage. However, further study is needed to determine the precise mechanism underlying the inhibitory activity of STK295900 on Top 1 and Top 2.

Basically, G_2_ arrest is regulated through the control of Cdk1 activity, which is regulated at multiple levels. In addition to association with cyclin B, the Cdk1 complex is activated by phosphorylation at T161 by Cdk-activating kinase (CAK). However, the cyclin B/Cdk1 complex is kept inactive by phosphorylation of Cdk1 at T14 and Y15 by Myt1 and Wee1, respectively [20], [44], [45]. Therefore, the activity of Cdk1 is regulated by the balance between the inhibitory kinases and the activating Cdc25 phosphatases that remove phosphates from T14 and Y15 for a timely control of G_2_/M transition [46]. Fig. 3A demonstrated that STK295900 induced G_2_ arrest was not associated with Wee1- and Myt1-mediated inhibitory phosphorylation of Cdk1 on T14 and Y15. However, further investigation is required to fully elucidate the mechanism of G_2_ arrest induced by STK295900.

Collectively, comparison of STK295900 with camptothecin, etoposide, and Hoechst 33342 for their growth inhibitory effects indicated that STK295900 is more cytotoxic to cancer cell lines than to normal cell lines (Table 2). STK295900 and the other symmetric bibenzimidazole derivatives are suggested to have potentials to be developed as anticancer agents.
