# Correction: STK295900, a Dual Inhibitor of Topoisomerase 1 and 2, Induces G_2_ Arrest in the Absence of DNA Damage

**DOI:** 10.1371/annotation/c5f432f6-1e92-42c0-8727-7ae972215d6f

**Published:** 2013-06-07

**Authors:** Sun-Ok Kim, Krisada Sakchaisri, N. R. Thimmegowda, Nak Kyun Soung, Jae-Hyuk Jang, Young Sang Kim, Kyung Sang Lee, Yong Tae Kwon, Yukihiro Asami, Jong Seog Ahn, Raymond Leo Erikson, Bo Yeon Kim

The third author's name was spelled incorrectly. The correct name is: N.R. Thimmegowda.

The correct citation is: Kim S-O, Sakchaisri K, Thimmegowda NR, Soung NK, Jang J-H, et al. (2013) STK295900, a Dual Inhibitor of Topoisomerase 1 and 2, Induces G2 Arrest in the Absence of DNA Damage. PLoS ONE 8(1): e53908. doi:10.1371/journal.pone.0053908 

